# Clinical and Complement Long-Term Follow-Up of a Pediatric Patient with C3 Mutation-Related Atypical Hemolytic Uremic Syndrome

**DOI:** 10.1155/2018/3810249

**Published:** 2018-12-18

**Authors:** Anna Bjerre, Grethe Bergseth, Judith Krey Ludviksen, Arne Stokke, Vidar Bosnes, Diana Karpman, Tom Eirik Mollnes

**Affiliations:** ^1^Division of Pediatric and Adolescent Medicine, Oslo University Hospital, Oslo, Norway; ^2^Research Laboratory, Nordland Hospital, Bodø, Norway; ^3^Department of Pediatrics, Nordland Hospital, Bodø, Norway; ^4^Department of Immunology, Section of Medical Immunology, Oslo University Hospital Ullevaal, Oslo, Norway; ^5^Department of Pediatrics, Clinical Sciences Lund, Lund University, Lund, Sweden; ^6^Faculty of Health Sciences, K.G. Jebsen TREC, University of Tromsø, Tromsø, Norway; ^7^Department of Immunology, Oslo University Hospital, University of Oslo, Oslo, Norway; ^8^Centre of Molecular Inflammation Research, Norwegian University of Science and Technology, Trondheim, Norway

## Abstract

We report a pediatric patient with atypical hemolytic uremic syndrome due to a C3 gain-of-function mutation diagnosed in infancy. She was treated from the start with a constant dose of 300 mg eculizumab every second week from the onset and followed by routine complement analyses for six years. Her complement system was completely inhibited and the dose interval was prolonged from 2 to 3 weeks without alteration of the dose and the complement activity continued to be completely inhibited. Blood samples taken immediately before, immediately after, and between eculizumab doses were analyzed for eculizumab-C5 complexes and percentage of total complement activity, using the Wieslab® test, and compared to a pool of sera from 20 healthy controls. The patient exhibited complete complement inhibition at all three time-points and had no free circulating C5 suggesting there was complete binding to eculizumab. She has now been treated for six years with full complement blockade. We suggest therefore that analysis of complement activity using the Wieslab® test is useful for evaluating the effect of eculizumab when dose intervals are prolonged.

## 1. Introduction

Atypical hemolytic uremic syndrome (aHUS) is a thrombotic microangiopathy characterized by platelet consumption, nonimmunological hemolysis, and renal injury [1]. It is well established that dysregulation of the alternative complement pathway is involved in 60-70% of all cases with loss-of-function mutations in complement regulators such as factor H, factor I, and membrane cofactor protein (MCP/CD46), or gain-of-function mutations in C3 or factor B [2]. In addition, autoantibodies to factor H can lead to factor H dysfunction and aHUS [3]. Since 2009, the humanized monoclonal anti-C5 antibody eculizumab has been the recommended drug for treatment and there is an international consensus that pediatric aHUS patients should be treated with eculizumab [2, 4] except for cases associated with autoantibodies. So far, the optimal dose of eculizumab and monitoring is not well defined and the treatment protocol dose is strictly categorized to weight and dose intervals. This issue is problematic due to the exuberant cost of the treatment [5] and therefore prolonging dose intervals could be cost-effective. We describe a pediatric patient with a C3 gain-of-function mutation and the effect of treatment with a constant dose of eculizumab (300 mg every three weeks) for six years with emphasis on the clinical course and the importance of complement analyses during follow-up for optimal evaluation of treatment effect.

## 2. Case Report

A 9-month-old girl was admitted to her local hospital because of increasing pallor and lethargy in conjunction with an upper airway tract infection. She had no diarrhea. On admission she had hematuria and blood tests showed hemoglobin 8.2 g/L (reference value 11.0-15.5 g/dL), platelet count 87 x 10^9^/L (150-450 x 10^9^/L), leukocytes 17 x 10^9^/L (5-15 x 10^9^/L), and creatinine 178 *μ*mol/L (20-30 *μ*mol/L). Due to gradual deterioration of her clinical condition, decreased diuresis, and worsening laboratory findings, hemolytic uremic syndrome (HUS) was suspected. She was transferred to Oslo University Hospital to undergo dialysis. On admission, blood tests confirmed the initial findings: anemia, thrombocytopenia, and kidney failure. Additional blood values showed lactate dehydrogenase (LDH) 2115 U/L (180-430 U/L), bilirubin 18 *μ*mol/L (5-25 *μ*mol/L), creatinine 193 *μ*mol/L, albumin 31 g/L (36-48 g/L), haptoglobin < 0.10 g/L (0.4-2.1 g/L), and C3 0.5 g/L (0.7-2.00 g/L). Schizocytes were present in the peripheral blood smear. Low C3 supported the suspected atypical HUS diagnosis. Combined treatment was initiated with plasma exchange (PE) and hemodialysis was initiated, with a good clinical response and normalization of hematology and renal function. Thereafter, during the following months, she developed three relapses, which all resolved after PE. A gain-of-function C3 mutation previously described in aHUS was identified in exon 14: R592W (R570W without the signal peptide) [6].

Use of the chimeric IgG2/4 monoclonal anti-C5 antibody eculizumab had recently been introduced [4]. Eculizumab treatment was started according to guidelines when the genetic results supported the diagnosis. Her weight at the start of treatment was 9.3 kg, with a starting dose of 300 mg in the first week, followed by another dose 2 weeks later and thereafter every third week until she reached 10 kg in weight. At a weight of 20 kg, it is recommended that the dose is increased to 600 mg every second week. Based on the complete inhibition of complement activity, we continued treatment with 300 mg every third week and followed the complement activity carefully. Complement activity assayed before each dose has shown complete suppression of the alternative and classical pathway Wieslab® assays, using the C5 dependent C5-9 complex as a readout (Complement screen kit, EuroDiagnostica, Malmö, Sweden), suggesting that the given dose was adequate during the entire six-year follow-up period (Table 1).

There have been no relapses of aHUS since the start of eculizumab treatment. At seven years of age, the patient has normal development, normal growth with a weight of 27 kg (50th percentile), and normal blood pressure. At the most recent visit, the laboratory hematology and biochemical values remained normal, and glomerular filtration rate measured by iohexol clearance was 84 ml/min/1.73m^2^. Urinalysis was normal with no hematuria or proteinuria.

The routine complement analyses are presented in Table 1. The low C3 values observed throughout the 6-year follow-up are consistent with continuous C3 consumption, due to the gain-of-function mutation. Importantly, this selective continuous increased C3 activation does not seem to affect her clinical or pathophysiologic condition adversely. This is consistent with blocking the terminal complement pathway with activation of C5 being responsible for the pathogenesis of this disease [7].

Complement activity was completely inhibited during the 6-year follow-up, indicating that a sufficient amount of eculizumab was administered even if the dose interval was prolonged to every third week, instead of every second week as recommended. In addition, the dose was not increased to 600 mg when reaching 20 kg as recommended by guidelines. Further on, we investigated in more detail the degree of C5 blockade and the complement activity in the course of one treatment interval. Three samples were collected for this purpose (Table 1): (1) 1 hr before infusion, (2) 1 hr after infusion, and (3) between two infusions of eculizumab (7 days after and 15 days before the next infusion); eculizumab-C5 complexes (E-C5) were quantified in an ELISA assay as previously described [8]. Briefly, the plate was coated with a monoclonal anti-human C5, incubated with serum samples (a source containing E-C5 complexes) followed by conjugated mouse anti-human IgG4 antibody for detection of eculizumab bound to C5.

**Figure 1:**
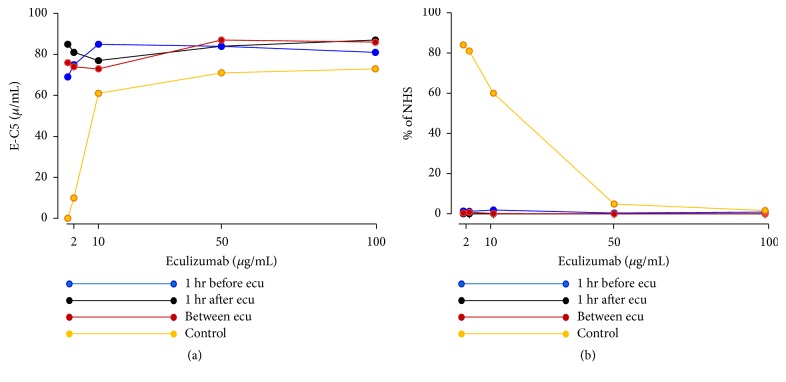
**Patient samples and control sera assayed for eculizumab-C5 (E-C5) complexes and total complement activity.** Three samples from the aHUS patient were examined: (1) sample obtained 1 hr before eculizumab infusion (“1 hr before ecu”), (2) sample obtained 1 hr after eculizumab infusion (“1 hr after ecu”), and (3) sample obtained 1 week after and 2 weeks before eculizumab infusion (“between ecu”). Control sera were obtained from 20 healthy controls. (a) Samples were incubated with increasing concentrations of eculizumab (0-100 *μ*g/mL) and E-C5 complexes were measured (concentration is given in eculizumab-equivalent amounts). (b) Samples were incubated with increasing concentrations of eculizumab (0-100 *μ*g/mL) and classical pathway complement activation was measured by the Wieslab® assay, detected as % of a normal human standard sera by the kit, defined to contain 100% activity (% of NHS).

Three patient samples and a control pool of sera from 20 healthy adult donors were available with approval of the regional ethics committee and informed written consent from patient's guardians and the controls. Samples were incubated with increasing doses of eculizumab (0-100 *μ*g/mL). No changes in E-C5 concentrations were observed in the three patient samples, whereas the pooled control sera exhibited a dose-dependent increase in E-C5 from zero to the levels corresponding closely to the patient samples, indicating that a maximum of E-C5 complexes were present in all three patient samples (Figure 1(a)). Thus, no free C5 was present to react with eculizumab.

We then investigated the effect of adding eculizumab (0-100 *μ*g/mL) on the complement activity as measured* in vitro *using the Wieslab test. Eculizumab blocks cleavage of C5 to C5a and C5b, thus inhibiting the formation of the C5b-9 complex in the assay. The three patient samples were completely negative from time zero, consistent with full blockade, whereas the control sera displayed a dose-response inhibition from normal activity to zero at eculizumab concentrations of 50-100 *μ*g/mL (Figure 1(b)).

## 3. Discussion

This report describes the follow-up of a child with aHUS and a C3 gain-of-function mutation. Even though the dosing interval of eculizumab initially was 300 mg every second week as recommended by the producer, we increased the interval to 3 weeks without dose alteration; we could demonstrate complete inhibition of the complement activity for more than 6 years paralleled by normal growth development and no signs of renal failure. The results suggest that a prolonged dosage regime can be applied with stringent follow-up of the complement blockade.

The doses and intervals of eculizumab administration are still not established. Recently an international consensus report suggested monitoring the effect of eculizumab treatment by global complement activity with a target of classical and alternative pathway activity < 10% measured by the traditional CH50 and AP50 red cell lysis models [9]. Using a similar modified approach, Ardissino et al. recently published their experience in reducing eculizumab doses to a targeted CP activity <30%, using the Wieslab® test [10]. This is only a modest blockade and might not be sufficient, and the long-term outcome of this approach is, as yet, not known. We have previously evaluated the effect of eculizumab by investigating the sensitivity of the Wieslab® test in patient samples and* in vitro* and found it to be highly sensitive, at a level of 1% of C5 present in plasma [11].

Several tests have been recently developed for the detection of complement activation, and the specific assays that are preferable for the follow-up of eculizumab-treated patients remain to be defined, as previously discussed [12]. This report suggests that the Wieslab® assay is useful for determining that sufficient concentrations of eculizumab have been administered to totally block complement activation. The special investigation of adding eculizumab to samples over a 3-week period supports the assay to be reliable with respect to detecting full blockade of the complement activity. Regular testing of complement activity may enable prolonged dose intervals and could be implemented in future individualized therapy. This can reduce the cost of therapy markedly. Notably, since only a small amount of complement is required to lyse red cells, as demonstrated in paroxysmal nocturnal hemoglobinuria, it is highly likely that the goal in complement therapy also in aHUS should be to block C5 completely, as was achieved in the present patient over a 6-year follow-up period.

In conclusion, we document the importance of investigating blockade of the complement system during follow-up of eculizumab treatment. In patients with aHUS, an evident appropriate blockade, it is possible to prolong dosage intervals, await dose increases, and thereby reduce the cost of therapy. Complement assays have been shown to be increasingly important for the investigation of the pathophysiology of aHUS. We emphasize the importance of using a standardized assay to detect the effect of eculizumab on complement inhibition, and propose these assays to be used for individualized treatment of these patients.

## Figures and Tables

**Table 1:** Complement analyses of the aHUS patient presented in this study.

Time of blood sampling (reference value)	C3^1^ (g/L) (0.70-2.00)^4^	C4^1^ (g/L) (0.10-0.50)	CP^2^ (%) (>40)	AP^2^ (%) (>10)	sC5b-9 (ng/mL)^3^ (<300)
2011, June Pre-treatment^5^	0.51	0.17	96	78	n.d.^6^
2011, June Post-eculizumab	0.48	0.13	<1	<1	140
2011, August	0.47	0.11	<1	<1	196
2015, May	0.54	0.16	3	<1	189
2016, May	0.47	0.16	2	<1	134
2017, January 5th^7^	0.44	0.12	<1	<1	106
2017, January 10th^8^ Pre-eculizumab	0.43	0.11	1	<1	n.d
2017, January 10th^8^ Post-eculizumab	0.46	0.12	<1	<1	n.d

^1^C3 and C4 were quantified in the routine hospital laboratory using nephelometry

^2^CP (classical pathway) and AP (alternative pathway) were quantified using the Wieslab® Complement screen kit (Euro Diagnostica AB, Malmö, Sweden). The reference ranges are given in % referred to 100% activity in normal human serum. These are based on screening for complement deficiencies. For detection of complete C5 blockade the values should be close to zero (< 3-5%).

^3^The soluble terminal C5b-9 complex was quantified using the sC5b-9 ELISA kit from Quidel, CA.

^4^Reference ranges are given in parenthesis.

^5^Before the first infusion of eculizumab.

^6^n.d. = not determined (sample not available).

^7^Sample obtained 7 days after and 15 days before eculizumab infusion.

^8^Samples obtained 1 hr before and 1 hr after eculizumab infusion.
